# Recently diagnosed rheumatoid arthritis patients benefit from a treat-to-target strategy: results from the DREAM registry

**DOI:** 10.1007/s10067-016-3191-3

**Published:** 2016-02-06

**Authors:** Laura M. M. Steunebrink, Harald E. Vonkeman, Peter M. ten Klooster, Monique Hoekstra, Piet L. C. M. van Riel, Mart A. F. J. van de Laar

**Affiliations:** Arthritis Center Twente, Department of Rheumatology, Medisch Spectrum Twente, PO BOX 50 000, 7500 KA Enschede, The Netherlands; Department Psychology, Health & Technology, University of Twente, Enschede, The Netherlands; Department of Rheumatology, Isala, Zwolle, The Netherlands; Department of Rheumatology, Bernhoven, Uden, The Netherlands

**Keywords:** Clinical practice, Early rheumatoid arthritis, ESR, Predictors, Remission, Treat-to-target

## Abstract

Despite considerable evidence on the efficacy and safety of early aggressive treat-to-target (T2T) strategies in early rheumatoid arthritis (RA), a proportion of patients still fail to reach remission. The goal of this study is to examine remission rates and predictors of remission in a real life T2T cohort of consecutive patients with a recent diagnosis of RA. Baseline demographics, clinical, laboratory and patient-reported variables and 1-year follow-up disease activity data were used from patients with early RA included in the DREAM remission induction cohort II study. Survival analyses and simple and multivariable logistic regression analyses were used to examine remission rates and significant predictors of achieving remission. A total of 137 recently diagnosed consecutive RA patients were available for this study. During the first year after inclusion, DAS28 remission was achieved at least once in 77.2 % of the patients and the median time to first remission was 17 weeks. None of the examined baseline variables were robustly associated with achieving remission within 1 year and in the multivariable analysis only lower ESR (*p* = 0.005) remained significantly associated with achieving fast remission within 17 weeks. During the first year of their disease a high proportion of recently diagnosed RA patient achieved remission, with only a small percentage of patients needing bDMARD therapy. Combined with the absence of baseline predictors of remission, this suggests that clinicians in daily clinical practice may focus on DAS28 scores only, without needing to take other patients characteristics into account.

## Introduction

Rheumatoid arthritis (RA) is a clinical diagnosis (or phenotype), considered to be the result of unidentified causes or insufficiently known pathophysiological malfunctions. Many conventional synthetic Disease-modifying anti-rheumatic drugs (csDMARDs) and biologicals (bDMARDs) are presently available and new treatment options and strategies have dramatically reduced the severity and impact of RA [1]. Early intensive combination therapies, aimed at achieving remission, have shown especially good clinical outcomes [2–4].

Early diagnosis and treatment of RA may constrain the progression of this disease. Since there is no cure for RA yet, the most important goal is to achieve stable remission as soon as possible [5–7]. Despite considerable evidence from clinical trials on the efficacy and safety of treatment strategies aiming at inducing remission, also known as treat-to-target (T2T), a relevant proportion of RA patients in daily clinical practice still fail to become free of signs and symptoms [8–13].

In recent years, the T2T approach has also been studied in observational studies such as the DREAM remission induction registry [3, 14], showing that remission is also an achievable goal in clinical practice. Ideally, this is achieved by a highly successful treatment strategy that is suitable for all RA patients in daily clinical care. If not all patients can be treated successfully, we may need personalized approaches and identification of those individual characteristics pointing to specific successful treatments [14, 15]. This personalized medicine approach has resulted in the search of predictors in genetic and other biomarkers [8, 16–18]. Unfortunately, until now major breakthroughs in predicting remission are lacking.

The DREAM remission induction cohort has demonstrated that T2T can be successfully implemented in a real life clinical setting. However, the question remains whether the T2T strategy fits all RA patients [13]. Therefore, the aim of this study is to examine remission rates and predictors of remission in a real life cohort of consecutive patients with a recent diagnosis of RA.

## Materials and methods

### Patients

This study used data from the ongoing DREAM (Dutch Rheumatoid Arthritis Monitoring) remission induction cohort II, an observational, multicenter cohort that was established in 2012 to evaluate the effects of a T2T strategy of initial combination therapy of methotrexate (MTX) and hydroxychloroquine (HCQ) aimed at quickly achieving a state of remission (DAS28 < 2.6) in early RA patients. Adults ≥ 18 years with a clinical diagnosis of RA and a disease duration < 1 year were enrolled in our study. Consecutive patients from two hospitals in The Netherlands, Medisch Spectrum Twente in Enschede and Isala in Zwolle, were included as soon as they had been diagnosed with RA. The treatment strategy is in line with clinical practice and complies with the current guidelines for the treatment of RA. Exclusion criteria were the use of prednisolone ≥ 10 mg/day and/or previous or current treatment with DMARDs. The ethics committees of the participating hospitals determined, in accordance with the relevant law in the Netherlands, that no ethical approval was required because all data were collected in the course of regular daily clinical practice. Nonetheless, patients were fully informed and prior informed consent was obtained from each patient.

### Treat-to-target protocol

Patients were evaluated at 0, 2, 4, and 6 months and every 3 months thereafter. Treatment advice consisted of an initial combination therapy of MTX 20 mg/week and HCQ 200 mg twice daily. As a bridging therapy, an optional intramuscular triamcinolone injection up to a maximum dosage of 120 mg could be given. After 1 month MTX dosage was increased to 25 mg/week, independent of disease activity. All patients who started MTX also received folic acid on the second day after MTX. After 2 months, in the case of persistent disease activity (DAS28 ≥ 2.6), MTX dosage was further increased to 30 mg/week and an extra optional intramuscular triamcinolone injection could be administered. After 4 months, in the case of moderate to high disease activity (DAS28 ≥ 3.2), a tumor necrosis factor (TNF) inhibitor (adalimumab, etanercept, or infliximab) was added. In cases of low disease activity (2.6 < DAS28 < 3.2), the attending rheumatologist could choose between adding sulphasalazine (SSZ) at 2000–3000 mg/day or an additional intramuscular triamcinolone injection.

Disease activity was assessed with the DAS28 during each subsequent visit. If remission (defined as DAS28 < 2.6) had not been achieved, treatment was intensified. However, if the patient was in DAS28 remission, medication was not changed. In case of sustained remission (≥6 months), medication was progressively reduced and eventually discontinued according to a predetermined tapering schedule. In the case of disease flare (DAS28 ≥ 2.6), the most recent medication or medication dosage that was shown to be effective was restarted, and treatment could subsequently be intensified. In response to clinical indications, the attending rheumatologist was free to diverge from the medication schedule at any time.

#### Measures

At each assessment, data were collected on various core set measures, including measures of disease activity, health related quality of life, physical functioning, and laboratory measures. Disease activity was assessed by trained rheumatology nurses using the disease activity score for 28 joints (DAS28), consisting of 28 swollen and tender joint count, the erythrocyte sedimentation rate (ESR), and a 100 millimeter visual analog scale (VAS) on general health (where 0 = “very good” and 100 = “very bad”) [19]. The Health Assessment Questionnaire Disability Index (HAQ-DI) was used to assess physical function [20]. Furthermore, patients rated their pain on a 100 millimeter VAS (0 = “no pain” and 100 = “unbearable pain”) and completed the 36 items of the Short Form Health Survey (SF-36) in order to assess their current physical and mental health status [21].

### Statistical analysis

Missing values for ESR (total 5 %, baseline 0.7 %) and for CRP (total 0.8 %, baseline 0 %) were imputed based on age, sex, swollen and tender joint counts, and ESR, or CRP using single imputation by applying the expectation-maximization method. Descriptive statistics for normally distributed variables or categorical variables were reported as frequencies, percentages, means and standard deviations (mean ± SD). If variables were not normally distributed, the median with the corresponding interquartile range (IQR) was reported. Kaplan-Meier survival analysis was performed to examine DAS28 remission rates and to compute the time point at which 50 % of the patients had achieved their first DAS28 remission (DAS28 < 2.6). Next, univariate logistic regression analyses were performed to examine associations between baseline variables and remission at the median survival time or within 1 year. Continuous predictors were evaluated for the suitability of the linearity assumption by plotting each variable against the logit of probability. Variables found to be related marginally (*p* < 0.20) to DAS28 remission at 17 weeks or 52 weeks in the univariate analysis were entered into multivariable logistic regression analysis as independent variables. Results were expressed as odds ratios (ORs) with 95 % confidence intervals (CIs). The final model was tested for goodness of fit using the Hosmer and Lemeshow test. The explained variance of the model was examined using Nagelkerke’s pseudo *R*^2^. All statistical calculations were performed using version 22 of the SPSS statistical package for Windows.

## Results

### Baseline characteristics

Table 1 summarizes the baseline characteristics of the total patient group and stratified by patients achieving remission or not at 6 months. A total of 137 patients were included. Patients had active disease at baseline, as demonstrated by high DAS28 scores and pain scores. Patients were, on average, 59 years old and the majority was female. Characteristics were similar between those that did or did not achieve remission at 6 months.

**Table 1:** Baseline characteristics

Characteristic	Score range of measure	Total group (*n* = 137)	Remission at 6 months (*n* = 86)	No remission at 6 months (*n* = 51)
Female sex, *n* (%)	–	82 (59.9)	48 (55.8)	17 (33.3)
Age, mean ± SD years	–	58.9 ± 13.5	59.0 ± 12.8	58.9 ± 14.9
DAS28–ESR, mean ± SD	0–10	4.9 ± 1.2	4.7 ± 1.2	5.2 ± 1.2
DAS28–CRP, mean ± SD	0–10	4.5 ± 1.2	4.4 ± 1.2	4.7 ± 1.2
ESR (mm/h), median (IQR)	0–140	29.0 (15.0–46.0) (*n* = 136)	24.5 (12.8–38.8)	35.0 (18.0–53.0) (*n* = 50)
CRP (mg/l), median (IQR)	0–999	12.0 (4.0–26.5)	11.5 (4.0–27.0)	13.0 (5.0–26.0)
Anti-CCP positive, *n* (%)	–	83 (61.0) (*n* = 136)	54 (62.8)	29 (56.9) (*n* = 50)
RF positive, *n* (%)	–	80 (58.4)	48 (55.8)	32 (62.7)
Number of SJC, median (IQR)	0–28	5.0 (2.0–10.0)	5.5 (2.0–10.0)	4.0 (2.0–10.0)
Number of TJC, median (IQR)	0–28	4.0 (2.0–11.0)	3.5 (1.8–1.3)	5.0 (2.0–11.0)
HAQ-SDI, mean ± SD	0–3	0.9 ± 0.7 (*n* = 90)	0.9 ± 0.6 (*n* = 63)	0.9 ± 0.8 (*n* = 27)
VAS well-being, mean ± SD	0–100	51.9 ± 26.3	50.5 ± 25.2	54.3 ± 28.2
VAS pain, mean ± SD	0–100	58.8 ± 22.3 (*n* = 75)	60 ± 21.9 (*n* = 46)	56.8 ± 23.1 (*n* = 29)
SF36-PCS, mean ± SD	0–100	37.3 ± 9.2 (*n* = 88)	37.6 ± 9.0 (*n* = 62)	36.7 ± 9.9 (*n* = 26)
SF36-MCS, mean ± SD	0–100	44.8 ± 11.9 (*n* = 88)	44.9 ± 13.1 (*n* = 62)	44.6 ± 8.3 (*n* = 26)
BMI, kg/m^2^, mean ± SD	–	26.1 ± 4.0 (*n* = 128)	26.1 ± 3.7 (*n* = 80)	26.0 ± 4.4 (*n* = 48)

*DAS28* disease activity score in 28 joints, *ESR* erythrocyte sedimentation rate, *CRP* C-reactive protein, *TJC* tender joint count, *SJC* swollen joint count, *HAQ-SDI* Health Assessment Questionnaire disability index (standard scoring), *SF-36* Short Form 36 health survey (version 2), *PCS* physical component summary, *MCS* mental component summary, *BMI* body mass index, *RF* rheumatoid factor, *Anti-CCP* anti-cyclic citrullinated peptide

### Time to reach first remission

**Fig. 1:**
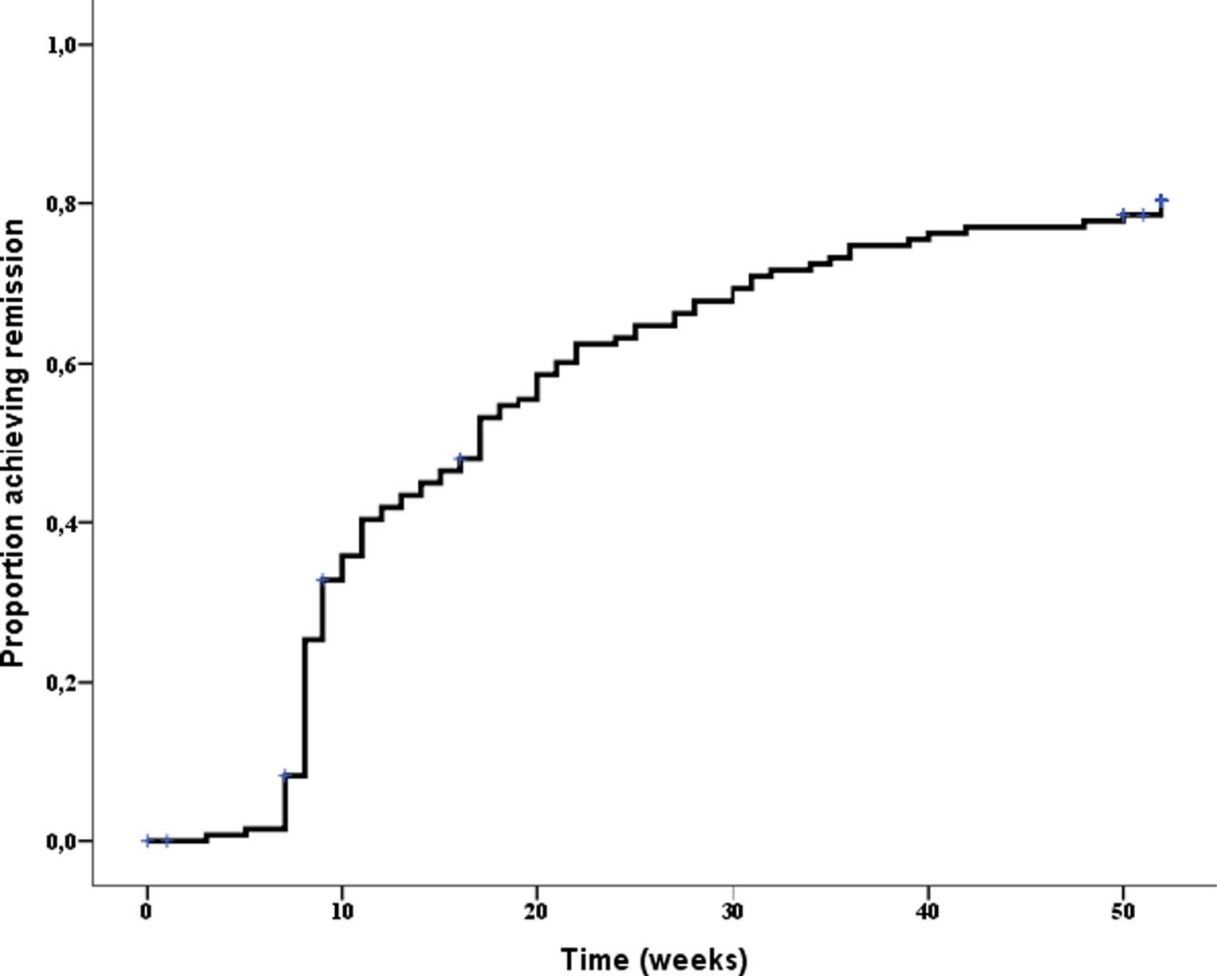
Kaplan-Meier curve for time to reach first remission after a 1-year follow-up in early RA patients

After 1 year of follow-up, remission (DAS28 < 2.6) was achieved at least once in 77.2 % of the patients and the median time to first remission was 17.0 (95 % CI: 13.0–20.0) weeks (Fig. 1). A total of 71 patients (51.8 %) achieved remission within 17 weeks.

### Univariate associations with remission within 17 and 52 weeks

Except for the baseline DAS28 score, none of the independent variables violated the assumption of linearity. Baseline DAS28 scores were dichotomized in high disease activity (>5.1) versus low and moderate disease activity. None of the examined baseline variables were significantly associated with achieving remission within 1 year (Table 2) and only lower HAQ-DI scores were marginally predictive of 1-year remission. Therefore, no multivariable model was tested for remission within 52 weeks. Lower ESR (*p* = 0.007), male gender (*p* = 0.057), and fewer tender joints (*p* = 0.175) were significantly or marginally associated with achieving remission within 17 weeks. As these variables were not strongly intercorrelated (all correlations < 0.10), they were simultaneously entered into multivariable analysis.

**Table 2:** Univariate predictors of remission within 17 weeks and 52 weeks

	Remission within 17 weeks			Remission within 52 weeks		
Variable	OR	95 % CI	p	OR	95 % CI	*p*
High disease activity (DAS28 > 5.1)	0.575	0.287–1.151	0.118	0.596	0.266–1.338	0.210
BMI	1.023	0.937–1.117	0.614	0.947	0.856–1.048	0.292
ESR	0.979	0.965–0.994	0.007	0.992	0.977–1.007	0.280
CRP	0.996	0.985–1.007	0.460	1.000	0.987–1.012	0.940
Anti-CCP positive	0.775	0.397–1.514	0.456	0.761	0.348–1.663	0.494
RF	1.110	0.788–1.564	0.549	1.071	0.710–1.616	0.744
Female sex	0.508	0.253–1.019	0.057	0.775	0.338–1.779	0.548
Age	0.993	0.969–1.018	0.587	0.988	0.959–1.019	0.456
HAQ-DI	0.820	0.444–1.514	0.526	0.671	0.300–1.500	0.331
PCS	1.025	0.978–1.074	0.309	1.014	0.954–1.077	0.663
MCS	1.009	0.973–1.046	0.621	1.013	0.967–1.062	0.577
VAS general well-being	0.994	0.981–1.007	0.332	0.993	0.977–1.009	0.374
SJC	0.978	0.922–1.037	0.454	1.029	0.955–1.108	0.452
TJC	0.963	0.912–1.017	0.175	0.969	0.912–1.030	0.312

*DAS28* disease activity score in 28 joints; *ESR* erythrocyte sedimentation rate; *CRP* C-reactive protein; *TJC* tender joint count; *SJC* swollen joint count; *HAQ-SDI* Health Assessment Questionnaire disability index (standard scoring); *SF-36* Short Form 36 health survey (version 2); *PCS* physical component summary; *MCS* mental component summary; *BMI* body mass index; *RF* rheumatoid factor; *Anti-CCP* anti-cyclic citrullinated peptide

### Multivariate predictors of remission within 17 weeks

The resultant multivariable model (Table 3) showed an adequate fit to the data (Hosmer and Lemeshow test: χ^2^ (8) = 10.43 *p* = 0.24), but only lower baseline ESR remained significantly predictive (*p* = 0.005) of achieving remission within 17 weeks (Table 3). No other variables were found to be significantly associated with remission and the total explained variance was low (13 %).

**Table 3:** Multivariate predictors of remission within 17 weeks in early RA

	Patients in remission in ≤ 17 weeks		
Variable	OR	95 % CI	p
ESR	0.978	0.963 – 0.993	0.005
Female sex	0.519	0.251 – 1.074	0.077
TJC	0.959	0.905 – 1.017	0.163

Nagelkerke *R* ^2^ = 0.129. *ESR* erythrocyte sedimentation rate; *TJC* tender joint count

### Medication at 12 months

**Fig. 2:**
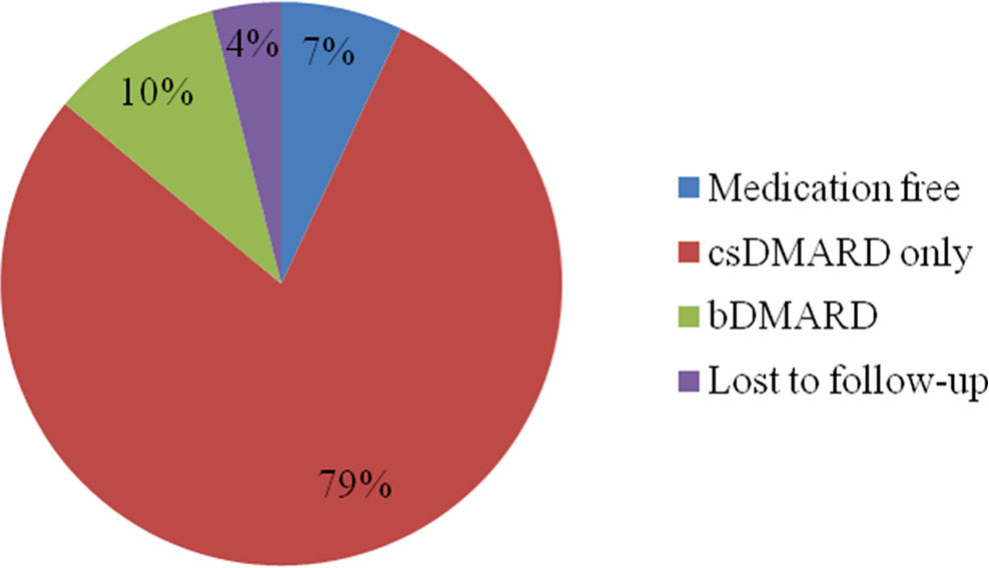
Medication percentages of patients with early RA at 12 months

At 12 months, 7.3 % (*n* = 10) of the patients were off medication entirely. The majority of the patients (54 %, *n* = 74) still used the initial combination medication of MTX + HCQ. In 9.5 % (*n* = 13) of the patients MTX monotherapy was used, while 13.9 % (*n* = 19) used another mono-csDMARD. MTX + bDMARD was used in 7.3 % (*n* = 10) of the patients at 12 months. Only one patient (0.7 %) was prescribed a bDMARD as mono therapy. The combination of two csDMARD and a bDMARD (MTX + HCQ + ADA) was used in 2.2 % of the patients (*n* = 3) and SSZ + HCQ was used in 1.5 % of the patients (*n* = 2). Five patients (3.6 %) were lost to follow-up (Fig. 2).

At baseline, 74 patients (54 %) received an intramuscular injection with triamcinolone. Furthermore, 30 patients (21.9 %) received triamcinolone injections between baseline and 12 months. Of these, 28 patients received one injection only 2 patients received 2 injections after baseline. Patients who received triamcinolone injections at baseline had significantly more swollen joints (8.0 (3.0–12.3) vs. 3.0 (2.0–6.0), *p* < 0.001) and more tender joint (6.0 (2.0–12.0) vs. 4.0 (1.0–8.0), *p* = 0.039) than patients who did not receive a triamcinolone injection. There were no significant differences in general health and ESR between those that received a baseline triamcinolone injection and those that did not.

## Discussion

This study demonstrated that almost 80 % of recently diagnosed RA patients rapidly achieved remission during the first year of their disease. Among a wide range of possible prognostic factors of achieving remission, no accurate predictors could be identified. This provides additional evidence that a T2T strategy aiming at remission is beneficial for recently diagnosed RA patients in clinical practice and should therefore be implemented widely.

The vast majority of the patients in the current study achieved the treatment target using csDMARDs only and only 10 % of patients needed to be prescribed a bDMARD within 12 months. In contrast to most previous T2T protocols, the protocol additionally allowed for optional intramuscular triamcinolone injections at the discretion of the treating rheumatologist. Interestingly, patients who received an injection at baseline generally had more swollen and tender joints than those who did not. However, there was no association with ESR or patient-reported general health. Apparently, the rheumatologists seemed to base their decision of using triamcinolone injections mostly on the number of affected joints and not on other indicators of disease activity. Future randomized studies could examine whether the number of affected joints is indeed a relevant indicator for the effectiveness of additional triamcinolone injections.

Fundamental, translational and clinical studies should ultimately lead to improvement of clinical care. Subsequent implementation in healthcare is often the real challenge; clinicians have to demonstrate that innovations are effective and safe, and contribute to increased health of the patient. The major strength of this study is the use of real life data from consecutive patients, recently diagnosed with RA, who were being treated according to a state of art T2T remission induction protocol. Limitations of the study are the relatively low number of patients (*n* = 137) and the absence of potential genetic predictors and other biomarkers. Also, in our study the follow-up period was only 1 year. Although this period of follow-up is not representative for the long patient journey of RA, until now successful early treatment is the best predictor of the future. However, longer follow-up is needed to establish whether current clinical remission rates prove sustainable [22].

The current study examined the prognostic value of a wide range of commonly available demographic and disease-related variables. Among these, no predictors were identified for (not) reaching remission. While some markers appeared to be associated with early remission at 17 weeks, only ESR remained significantly predictive in multivariable analysis. The finding that low ESR at baseline was associated with achieving DAS28 remission at 17 weeks is not surprising, as ESR is one of the components of the DAS28. Moreover, this finding is in line with earlier studies by Forslind et al. [23], who found that patients with high baseline DAS28 were less likely to achieve remission in all models and at all time points. Low ESR also showed a non-significant trend in the study by Vazquez et al. [24] and Burmester et al. [25] also found a univariate association with lower CRP, an acute phase reactant similar to ESR.

The finding that none of the potential predictors were significantly associated with achieving remission is in contrast with previous reports in early RA which have reported a variety of predictors ranging from sociodemographic characteristics, clinical and laboratory measures, to genetic markers. More specifically, female sex [23, 26, 27], older age [26, 28], RF positivity [17, 23, 29], and smoking [30] were previously found to be associated with fewer non-remission periods. The finding that none of these factors were predictive in the present study may be due to the very high proportion of patients achieving remission (almost 80 %) in the current T2T strategy where other studies have reported remission percentages ranging from 10–65 % [31].

In conclusion, the results of this study demonstrated that the current T2T strategy appears to be broadly applicable to all patients with recently diagnosed RA. Together with the absence of clear baseline predictors of remission, this suggests that clinicians in daily clinical practice may focus on DAS28 scores only, without needing to take other patients characteristics into account.

## References

[1] Donahue KE, Gartlehner G, Jonas DE (2008). Systematic review: comparative effectiveness and harms of disease-modifying medications for rheumatoid arthritis. Ann Intern Med.

[2] Ma MHY, Kingsley GH, Scott DL (2010). A systematic comparison of combination DMARD therapy and tumour necrosis inhibitor therapy with methotrexate in patients with early rheumatoid arthritis. Rheumatology (Oxford).

[3] Vermeer M, Kuper HH, Hoekstra M (2011). Implementation of a treat-to-target strategy in very early rheumatoid arthritis: results of the Dutch Rheumatoid Arthritis Monitoring remission induction cohort study. Arthritis Rheum.

[4] Vermeer M, Kuper HH, Moens HJB (2013). Sustained beneficial effects of a protocolized treat-to-target strategy in very early rheumatoid arthritis: three-year results of the Dutch Rheumatoid Arthritis Monitoring remission induction cohort. Arthritis Care Res (Hoboken).

[5] Gremese E, Salaffi F, Bosello SL (2013). Very early rheumatoid arthritis as a predictor of remission: a multicentre real life prospective study. Ann Rheum Dis.

[6] Schoels M, Knevel R, Aletaha D (2010). Evidence for treating rheumatoid arthritis to target: results of a systematic literature search. Ann Rheum Dis.

[7] Smolen JS, Aletaha D, Bijlsma JWJ (2010). Treating rheumatoid arthritis to target: recommendations of an international task force. Ann Rheum Dis.

[8] Gossec L, Dougados M, Goupille P (2004). Prognostic factors for remission in early rheumatoid arthritis: a multiparameter prospective study. Ann Rheum Dis.

[9] Scott DL, Symmons DP, Coulton BL, Popert AJ (1987). Long-term outcome of treating rheumatoid arthritis: results after 20 years. Lancet.

[10] Pope RM (1996). Rheumatoid arthritis: pathogenesis and early recognition. Am J Med.

[11] Shaver TS, Anderson JD, Weidensaul DN (2008). The problem of rheumatoid arthritis disease activity and remission in clinical practice. J Rheumatol.

[12] Van der Helm-van Mil AHM, Breedveld FC, Huizinga TWJ (2006). Aspects of early arthritis. Definition of disease states in early arthritis: remission versus minimal disease activity. Arthritis Res Ther.

[13] Jayakumar K, Norton S, Dixey J (2012). Sustained clinical remission in rheumatoid arthritis: prevalence and prognostic factors in an inception cohort of patients treated with conventional DMARDS. Rheumatology (Oxford).

[14] Schipper LG, Vermeer M, Kuper HH (2012). A tight control treatment strategy aiming for remission in early rheumatoid arthritis is more effective than usual care treatment in daily clinical practice: a study of two cohorts in the Dutch Rheumatoid Arthritis Monitoring registry. Ann Rheum Dis.

[15] Siemons L, Ten Klooster PM, Vonkeman HE, et al. (2013) Distinct trajectories of disease activity over the first year in early rheumatoid arthritis patients following a treat-to-target strategy. Arthritis Care Res (Hoboken). doi: 10.1002/acr.22175 10.1002/acr.22175 24106173

[16] Eberhardt K, Fex E (1998). Clinical course and remission rate in patients with early rheumatoid arthritis: relationship to outcome after 5 years. Br J Rheumatol.

[17] Verstappen SMM, van Albada-Kuipers GA, Bijlsma JWJ (2005). A good response to early DMARD treatment of patients with rheumatoid arthritis in the first year predicts remission during follow up. Ann Rheum Dis.

[18] Möttönen T, Hannonen P, Korpela M (2002). Delay to institution of therapy and induction of remission using single-drug or combination-disease-modifying antirheumatic drug therapy in early rheumatoid arthritis. Arthritis Rheum.

[19] Prevoo ML, van ’t Hof MA, Kuper HH (1995). Modified disease activity scores that include twenty-eight-joint counts. Development and validation in a prospective longitudinal study of patients with rheumatoid arthritis. Arthritis Rheum.

[20] Bruce B, Fries JF (2005). The Health Assessment Questionnaire (HAQ). Clin Exp Rheumatol.

[21] Ware JE, Sherbourne CD (1992). The MOS 36-item short-form health survey (SF-36). I. Conceptual framework and item selection. Med Care.

[22] Kuriya B, Xiong J, Boire G (2014). Earlier time to remission predicts sustained clinical remission in early rheumatoid arthritis—results from the Canadian Early Arthritis Cohort (CATCH). J Rheumatol.

[23] Forslind K, Hafström I, Ahlmén M, Svensson B (2007). Sex: a major predictor of remission in early rheumatoid arthritis?. Ann Rheum Dis.

[24] Vázquez I, Graell E, Gratacós J (2007). Prognostic markers of clinical remission in early rheumatoid arthritis after two years of DMARDs in a clinical setting. Clin Exp Rheumatol.

[25] Burmester GR, Ferraccioli G, Flipo R-M (2008). Clinical remission and/or minimal disease activity in patients receiving adalimumab treatment in a multinational, open-label, twelve-week study. Arthritis Rheum.

[26] Wolfe F, Hawley DJ (1985). Remission in rheumatoid arthritis. J Rheumatol.

[27] Harrison BJ, Symmons DP, Brennan P (1996). Natural remission in inflammatory polyarthritis: issues of definition and prediction. Br J Rheumatol.

[28] Pease CT, Bhakta BB, Devlin J, Emery P (1999). Does the age of onset of rheumatoid arthritis influence phenotype? A prospective study of outcome and prognostic factors. Rheumatology (Oxford).

[29] Mancarella L, Bobbio-Pallavicini F, Ceccarelli F (2007). Good clinical response, remission, and predictors of remission in rheumatoid arthritis patients treated with tumor necrosis factor-alpha blockers: the GISEA study. J Rheumatol.

[30] Van der Woude D, Young A, Jayakumar K (2009). Prevalence of and predictive factors for sustained disease-modifying antirheumatic drug-free remission in rheumatoid arthritis: results from two large early arthritis cohorts. Arthritis Rheum.

[31] Van der Kooij SM, Allaart CF, Dijkmans BA, Breedveld FC (2008). Innovative treatment strategies for patients with rheumatoid arthritis. Curr Opin Rheumatol.

